# Enhancing Radiopacity and Antibacterial Activity of Osteogenic Calcium Silicate Cement by Incorporating Strontium

**DOI:** 10.3390/jfb16120445

**Published:** 2025-11-28

**Authors:** Ting-Yi Chiang, Yu-Ci Lu, Chun-Cheng Chen, Shinn-Jyh Ding

**Affiliations:** 1 Department of Dental Technology and Materials Science, Central Taiwan University of Science and Technology, Taichung 406, Taiwan; 2 Institute of Oral Science, Chung Shan Medical University, Taichung 402, Taiwan; 3 School of Dentistry, Chung Shan Medical University, Taichung 402, Taiwan; 4 Department of Stomatology, Chung Shan Medical University Hospital, Taichung 402, Taiwan

**Keywords:** bone cement, calcium silicate, strontium, radiopacifier, osteogenic activity, antibacterial activity

## Abstract

Calcium silicate-based cement is commonly used for bone repair and regeneration. Current research focuses on developing innovative antibacterial materials with radiopacity, which is essential for ensuring successful clinical outcomes in procedures like vertebroplasty and endodontic treatments. Strontium (Sr) has emerged as a powerful additive, stimulating bone formation and inhibiting bone resorption. In this study, we evaluated the impact of varying levels of Sr—5, 10, and 20 mol% (designated as CSSr5, CSSr10, and CSSr20) on critical attributes of bone cement, including radiopacity, setting time, in vitro bioactivity, antibacterial efficacy, and osteogenic activity. The findings indicated that as the Sr content increased, the setting time and radiopacity of the cement increased. Remarkably, the cement formulations containing over 10 mol% Sr achieved radiopacity values surpassing the 3 mm aluminum threshold mandated by ISO 6876:2001 standards. Furthermore, incorporating Sr significantly improved MG63 cell attachment, proliferation, differentiation, and mineralization, while also boosting antibacterial properties in a dose-dependent manner. After 48 h of inoculation with *E. coli* or *S. aureus*, the CSSr10 and CSSr20 cements showed a bacteriostatic ratio exceeding 1.7 or 2 times that of the control without Sr. In conclusion, the CSSr10 cement could be a promising bone filler, exhibiting favorable setting time, radiopacity, antibacterial ability, and osteogenic activity.

## 1. Introduction

Bone defects resulting from tumor resections, nonunion fractures, infections, or abnormal skeletal development can lead to significant skeletal dysfunction and disability, often leading to bone loss [1,2]. Restoring normal bone function and structure to address these defects is a critical clinical challenge. In response, synthetic bone grafts have been developed as alternatives to natural bone grafts. These artificial options aim to repair bone loss and promote bone regeneration using various scaffolds and bone cements [1,2,3,4,5]. When bone cement is injected into bone defects, it not only fills the shape of the defect cavity but also hardens into a solid form, unlike the bulk scaffold. This minimally invasive injectable material helps reduce operation time, minimizes damage to surrounding muscles and scar size, and alleviates postoperative pain, allowing patients to recover more quickly [6]. Hydraulic calcium silicate cements have garnered attention due to their exceptional capabilities in bone repair and regeneration [7,8,9]. Furthermore, these cements create a localized high pH environment, contributing to their partial antibacterial effect [10]. This attribute is critically important in light of the rising resistance of bacteria to antibiotics. As a result, numerous studies are actively exploring the integration of non-antibiotic antibacterial agents—whether inorganic or organic—further to enhance the antibacterial efficacy of bioactive bone cements, all while preserving their essential osteogenic properties [11,12].

An implanted material must be visible to ensure optimal contact with the repaired bone defect area during X-ray detection for effective post-monitoring [4,13]. Unfortunately, the inherently low radiopacity of calcium silicate cement renders it nearly indistinguishable from surrounding tooth and bone tissues [14,15]. This significant limitation in radiopacity hinders the clinical application of this cement and poses challenges in critical areas such as endodontics and vertebroplasty. To address this, incorporating radiopacifiers has become necessary to meet the rigorous radiopacity standards outlined in ISO 6876:2001: Dental root canal sealing materials, which mandates a minimum of 3 mm of Al. However, incorporating radiopacifiers like Bi_2_O_3_ and ZrO_2_ can negatively impact the physicochemical and biological properties of the bone cement [4,15]. In light of these challenges, strontium (Sr) has emerged as a promising alternative, offering the potential to achieve the desired radiopacity without compromising the essential properties of the cement [16,17,18].

Strontium is vital in maintaining bone homeostasis during remodeling due to its dual functions of promoting bone formation and reducing bone resorption [5,19,20,21,22,23]. Research conducted by Grynpa et al. demonstrated that administering low doses of Sr significantly increased the number of bone-forming sites and enhanced vertebral bone volume in rats [22]. Notably, this treatment did not negatively impact mineral profiles, bone mineral chemistry, or matrix mineralization, indicating its potential as a safe and effective option for improving bone health. With the aging population facing an escalating prevalence of osteoporosis, the importance of effective treatments cannot be overstated. Sr has been shown to help increase bone mass in osteoporosis patients, providing hope for improved outcomes [23]. Additionally, a study using a cancellous bone model from the ilium of rabbits found that Sr-containing hydroxyapatite (HA) cement showed promise as a potential treatment for vertebroplasty in osteoporotic fractures [13].

Sr shares physical and chemical similarity with calcium and has a protein-binding capacity in plasma or serum that resembles that of calcium [18]. This similarity has led to numerous studies incorporating Sr into calcium phosphate or calcium silicate-based materials [5,17,18,23,24,25,26]. For instance, Lode et al. found that Sr-substituted calcium phosphate bone cements can enhance the strength and opacity of hardened bone cements while also stimulating the proliferation and osteogenic differentiation of bone cells [18]. Similarly, Wang et al. demonstrated that a low concentration of Sr in bioactive glass improved the alkaline phosphatase (ALP) activity of bone marrow mesenchymal stem cells compared to Sr-free bioactive glass [24]. Animal experiments on rabbits have further validated these findings, revealing that Sr-containing HA bone cement integrates well within the maxilla and tibia [25]. Moreover, research by Wu et al. demonstrated that Sr-doped calcium silicate-based bioceramic scaffolds can facilitate the osteogenic differentiation of osteoporotic mesenchymal stem cells in vitro, with in vivo studies also indicating effective bone regeneration capabilities, including angiogenesis [26]. Additionally, Sr ions display antibacterial properties that depend on their concentration [27,28]. However, exploring Sr’s role in antibacterial activity has received less attention than silver. This underscored a vital area for further investigation, as the promising benefits of Sr-based materials could significantly advance biomedical applications.

From a clinical standpoint, enhancing the radiopacity, antibacterial properties, and osteogenic activity of calcium silicate cement is essential for improving patient outcomes. This study delved into the characteristics of Sr-incorporated calcium silicate cement, which was prepared using the sol–gel method. We systematically analyzed how Sr dopants influence the cement’s physicochemical properties, osteogenic activities, and antibacterial effectiveness.

## 2. Materials and Methods

### 2.1. Specimen Preparation

Tetraethyl orthosilicate (TEOS; Sigma-Aldrich, St. Louis, MO, USA), calcium nitrate tetrahydrate (Showa, Tokyo, Japan), and strontium nitrate (Alfa Aesar, Ward Hill, MA, USA) were utilized to prepare a powder using the sol–gel method. First, TEOS and absolute ethanol were added to 2 M HNO_3_ and stirred for two hours to ensure complete dissolution. Next, Ca(NO_3_)_2_·4H_2_O and/or Sr(NO_3_)_2_ were added, followed by an additional hour of stirring. Three different amounts of Sr were incorporated with molar ratios of 5%, 10%, and 20% for Sr/(Ca + Sr). The molar ratio of (Ca + Sr) to Si was 1:1. The sol solution was aged at 60 °C for one day and then dried at 120 °C for another day. The dried gel was subsequently heated in air to 800 °C at a heating rate of 10 °C/min for two hours to produce a powder, which was then ball-milled for 12 h using a Retsch PM 100 centrifugal ball mill (Hann, Germany). The sample codes for the resulting powders and cements were designated as “CS,” “CSSr5,” “CSSr10,” and “CSSr20,” representing the cement compositions with Sr/(Sr + Ca) ratios of 0%, 5%, 10%, and 20% mol%, respectively. The cement sample was prepared at a liquid-to-powder ratio of 0.45 mL/g. These cements were placed in a cylindrical Teflon mold to form specimens, which were then stored in an incubator at 100% relative humidity and 37 °C for one day to allow for setting, except for the setting time measurements.

### 2.2. Setting Time

According to ISO 9917-1:2003 (Dentistry—Water-based cements—Part 1: Powder/liquid acid-base cements), a 400 g Gillmore needle (1 mm in diameter) was used to assess the setting time of bone cement. After mixing the powder with water for one minute, the mixture was placed in a Teflon mold to form a 6 mm × 6 mm sample. The test procedure adhered to methods outlined in a previous study [15]. The average setting time was determined based on ten replicate specimens.

### 2.3. Phase Composition and Morphology

Samples were evaluated using X-ray diffractometry (XRD) with a Bruker D8 SSS (Karlsruhe, Germany) to investigate their phase composition. The analysis utilized Ni-filtered CuKα radiation and was performed at a scanning speed of 1°/min. The chemical structure was examined by Fourier transform infrared spectroscopy (FTIR; Bruker Vertex 80v, Ettlingen, Germany) in transmittance mode. The surface morphology was observed using a JEOL JSM-7800F field-emission scanning electron microscope (FESEM; Tokyo, Japan). The specimens were sprayed with a gold layer using a JFC-1600 coater from JEOL.

### 2.4. Diametral Tensile Strength

The strength evaluation was performed using an EZ-Test machine (Shimadzu, Kyoto, Japan) at a 0.5 mm/min loading rate. The following formula was used to calculate the strength value: strength = 2F/(πdt), where F represents the peak load (in Newtons), and d and t denote the diameter (in mm) and thickness (in mm) of the specimen, respectively. Each specimen measured 6 mm in diameter and 3 mm in thickness. The reported data represented the average of 15 measurements.

### 2.5. Porosity

The porosity of the cement specimens was measured using Archimedes’ method, based on a previous study that utilized ethanol as the test liquid [9]. The data presented are mean ± standard deviation from five independent measurements.

### 2.6. Radiopacity

The radiopacity of the cement was evaluated using the method outlined in ISO 6876: 2001, employing a Belray 096 Dental X-ray unit (Takara Belmont Corp., Osaka, Japan). The radiopacity of the cement specimen was measured in relation to the thickness of a standardized aluminum step wedge, which comprised 10 steps, each measuring 1 mm [15]. The gray data were digitized using ImageJ software. Three parallel experiments were conducted for each group.

### 2.7. In Vitro Bioactivity

Cement samples were immersed in simulated body fluid (SBF) solutions with a pH of 7.4 at 37 °C [29]. The pH levels were adjusted using hydrochloric acid and tris(hydroxymethyl)aminomethane. After predetermined soaking durations, the fifteen samples per group were either removed from the vial for strength measurements or dried at 60 °C for subsequent analysis of physicochemical properties.

### 2.8. Osteoblast Responses

The responses of human osteoblast-like cell line MG63 (BCRC 60279, Hsinchu, Taiwan) to the cement were assessed through direct culture. The cement sample was placed in a 48-well tissue culture plate and sterilized by soaking it in a 75% ethanol solution, followed by exposure to ultraviolet light for 2 h. Dulbecco’s modified Eagle medium (Gibco, Langley, OK, USA), supplemented with 10% fetal bovine serum (FBS; Gibco) and 1% penicillin/streptomycin solution (Gibco), was used for culturing the MG63 cells at a density of 5 × 10^3^ cells/mL. Cell attachment was evaluated at 6 and 12 h, while cell proliferation was assessed at 1, 3, and 7 days using the MTT (Sigma-Aldrich) assay. The ALP activity was measured at 7 and 14 days using the Takara TRACP & ALP assay kit (Shiga, Japan), per the manufacturer’s instructions. On the 7th and 14th day, Alizarin Red S staining was performed to analyze the mineralized matrix [9]. The absorbance was measured using a BioTek Epoch spectrophotometer (Winooski, VT, USA), with three measurements taken for accuracy.

### 2.9. Antibacterial Activity

Colonies of *Escherichia coli* (*E. coli*; ATCC 8739, Hsinchu, Taiwan) and *Staphylococcus aureus* (*S. aureus*; ATCC 25923, Hsinchu, Taiwan) in Bacto tryptic soy broth (Becton Dickinson, Sparks, MD, USA) were utilized to assess the antibacterial effectiveness of the cement. A bacterial suspension (10^7^ CFU/mL) was added to the cement, with culture durations of 3, 6, 12, 24, and 48 h, in accordance with a previous study [27]. The growth of bacteria without the cement was referred to as the negative control. The bacterial amounts on the samples were evaluated using an Alamar Blue assay (Invitrogen, Grand Island, NY, USA). The absorbance of the samples was measured with a BioTek Epoch spectrophotometer. The bacteriostatic ratio (%) was calculated using the equation: (absorbance of the negative control—absorbance of the cement sample)/absorbance of the negative control × 100%. Three measurements were taken to obtain an average of the data.

### 2.10. Statistical Analysis

One-way analysis of variance (ANOVA) and Scheffé’s multiple comparison testing were conducted to evaluate the significance of the differences between the measurement values. Results were considered statistically significant at a *p*-value of less than 0.05.

## 3. Results

### 3.1. Phase Composition and Morphology of Powders

**Figure 1:**
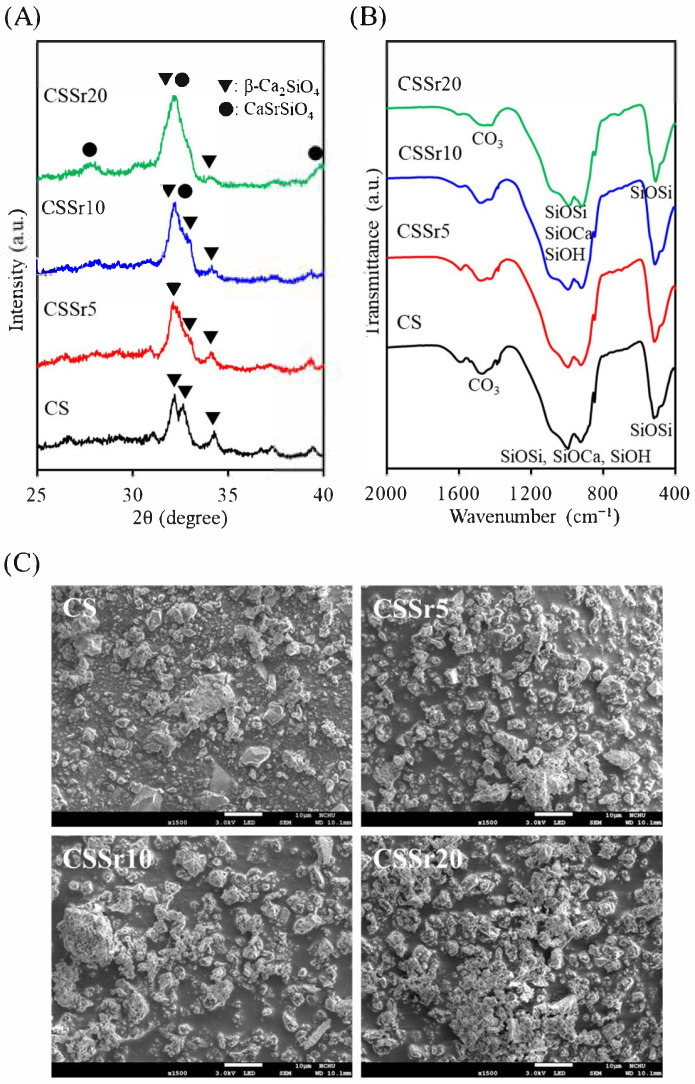
(**A**) XRD patterns, (**B**) FTIR spectra, and (**C**) SEM micrographs of the four powders.

Figure 1A displayed the XRD patterns of the prepared powders. The prominent diffraction peaks of the CS control at 2θ = 32.2°, 32.6°, and 34.2° were attributed to the β-Ca_2_SiO_4_ (β-dicalcium silicate) phase [9]. A broad diffraction peak around 30° was likely due to the presence of the amorphous SiO_2_ phase. When Sr was incorporated into the CS, the broadened peak observed in the 31–33° range may be associated with the overlap of the CaSrSiO_4_ (calcium strontium silicate) and β-Ca_2_SiO_4_ phases. Weak peaks at 27.7° and 39.7° were also linked to the CaSrSiO_4_ phase [30].

Figure 1B showed the FTIR spectra of the four powder samples in transmission mode, which were similar and consistent with the XRD results. The bands detected between 1500 and 1400 cm^−1^ likely correspond to the vibrational mode of the CO_3_ group, possibly resulting from atmospheric carbonation. The absorption band corresponding to SiO_4_ asymmetric stretching and Si–O–Ca bond formation spans a wide wavenumber range from 1200 to 900 cm^−1^, overlapping with the Si–OH asymmetric stretching observed from 980 to 900 cm^−1^. The band at approximately 520 cm^−1^ was related to the bending or rocking of Si–O–Si bonds [9].

In terms of morphology, the sinter-milled powder exhibited an irregular shape, with particle sizes ranging from 1 µm to 5 µm, as illustrated in Figure 1C. The particles exhibited a heterogeneous structure, comprising both porous and dense bodies. Incorporating Sr into the SiO_2_–CaO powder did not cause any significant changes in its morphology.

### 3.2. Physicochemical and Mechanical Properties of Cement

The setting time of the cement increased linearly with the higher Sr content in the cement powders, ranging from 17 ± 2 min for the control cement (CS) to 39 ± 2 min for the CSSr20 cement (Table 1), indicating a statistical difference (*p* < 0.05). Additionally, the radiopacity of the CS control was recorded at 1.9 mm Al (Table 1), which was below the recommended level of 3 mm Al. As expected, the radiopacity rose with increasing Sr content, with the CSSr20 showing a significantly higher value (*p* < 0.05) of 6.1 mm, which was clinically acceptable. Moreover, Table 1 illustrated the relationship between Sr content and diametral tensile strength or porosity of the cements. The strength values were in the range of 2.4–2.6 MPa, while the porosity ranged from 23% to 28%, indicating no significant difference (*p* < 0.05) among the four cement samples.

### 3.3. In Vitro Bioactivity

#### 3.3.1. Phase and Morphology

**Figure 2:**
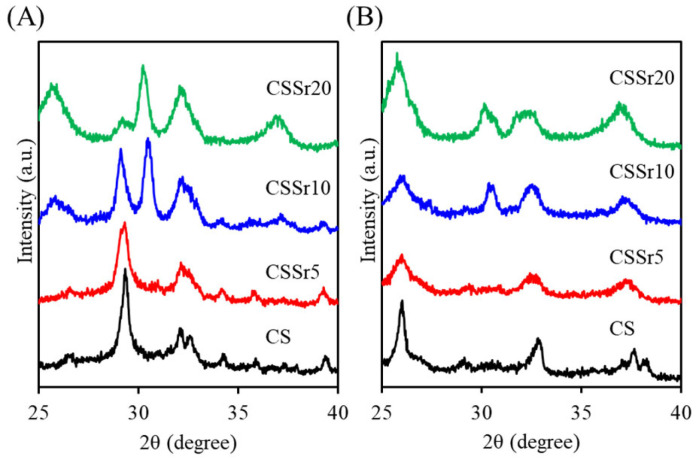
XRD patterns of the four cement samples (**A**) before and (**B**) after soaking in SBF for one day.

When the powder was mixed with water, the resulting hardened CS-based cements formed a new calcium silicate hydrate (C–S–H) peak at approximately 2θ = 29.4°. This peak was attributed to the hydration reaction of β-Ca_2_SiO_4_ (Figure 2A) [9]. Furthermore, some unreacted β-Ca_2_SiO_4_ remained present. In cement samples with a high Sr content, the C–S–H peak weakened, and new peaks appeared at 2θ = 25.6°, 30.2°, and 36.9°, likely due to the hydration reaction of CaSrSiO_4_. After soaking the samples in SBF for one day (Figure 2B), the XRD patterns of the CS control indicated an obvious decrease in the C–S–H hydration product, with a new peak 2θ = 26.0° emerging, which was associated with the precipitated apatite.

**Figure 3:**
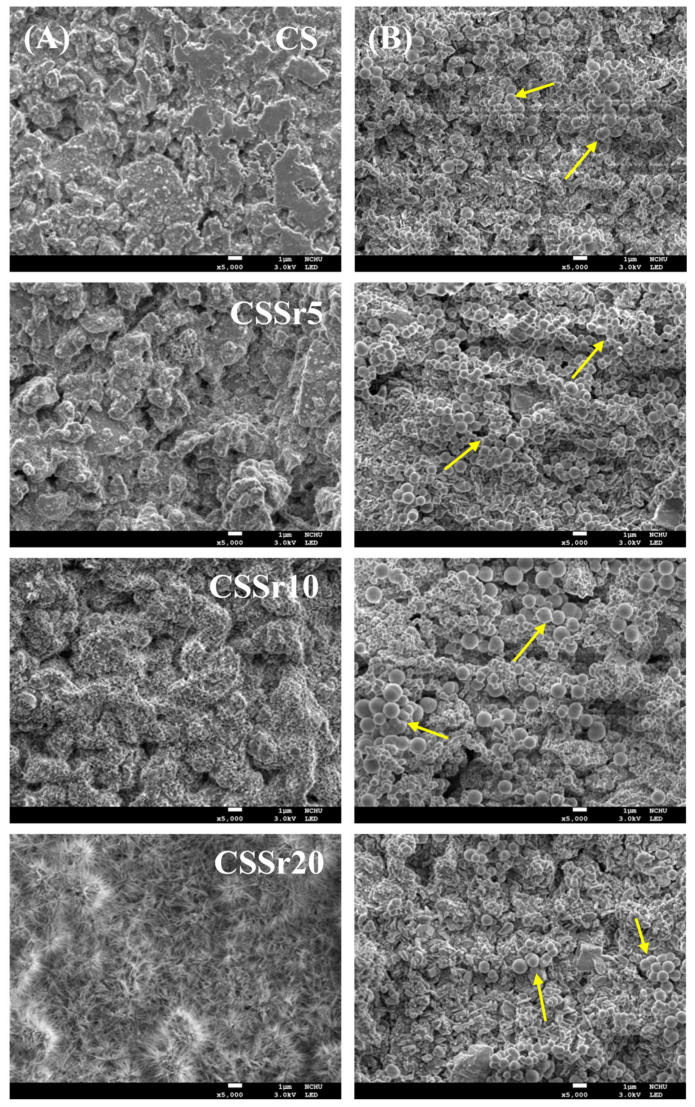
SEM micrographs of various cements (**A**) before and (**B**) after soaking in SBF for one day. The arrows indicate the precipitated spherulites.

The SEM micrographs of the various cements, both before and after soaking in SBF, were shown in Figure 3. The surface of the as-hardened CS cement primarily consisted of entangled particles and numerous micropores (Figure 3A). The lower Sr content cement exhibited a similar structure. However, the cement with a higher Sr content (CSSr20) displayed a fibril-like crystalline structure, likely due to the hydration reaction of CaSrSiO_4_. After one day of soaking in SBF (Figure 3B), the surface microstructure of all four cements changed significantly. The surfaces of all cement samples became covered with clusters of precipitated spherulites, as indicated by the arrows in the figures.

#### 3.3.2. Strength

**Figure 4:**
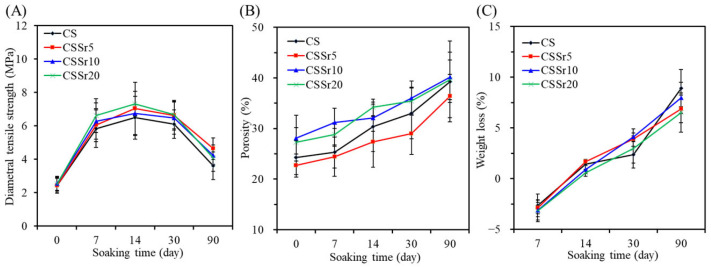
(**A**) Diametral tensile strength, (**B**) porosity, and (**C**) weight loss of various bone cements before and after soaking in an SBF solution for different time points.

When the cement samples were immersed in SBF, their diametral tensile strength gradually increased, followed by a decline (Figure 4A). For instance, in the CSSr10 group, the strength values were 6.3 MPa after 7 days, 6.7 MPa after 14 days, 6.4 MPa after 30 days, and 4.2 MPa after 90 days of immersion. These strength values were significantly higher (*p* < 0.05) than the initial strength of 2.6 MPa recorded on day 0. Furthermore, it is worth noting that no statistically significant differences were found among the four types of cement samples tested at the same soaking duration. After 90 days of immersion, the strength values were as follows: 3.6 MPa for CS, 4.6 MPa for CSSr5, 4.2 MPa for CSSr10, and 4.1 MPa for CSSr20 cement samples.

#### 3.3.3. Porosity and Weight Loss

The porosity of the cement samples increased over time, as shown in Figure 4B. Remarkably, this increase remains unaffected by the type of cement used. After a 90-day period, the porosity of the cement samples ranged from 36% to 40%. Figure 4C illustrates the impact of soaking time on the weight changes in the cement samples. On day 7, all four cement samples experienced a slight increase in weight. However, as immersion continued, the samples began to lose weight. By the 90-day point, this weight loss ranged from 6% to 9%. Importantly, statistical analysis revealed no significant differences (*p* > 0.05) in weight loss among the cement samples at comparable soaking times.

### 3.4. Osteoblast Responses

#### 3.4.1. Cell Growth

**Figure 5:**
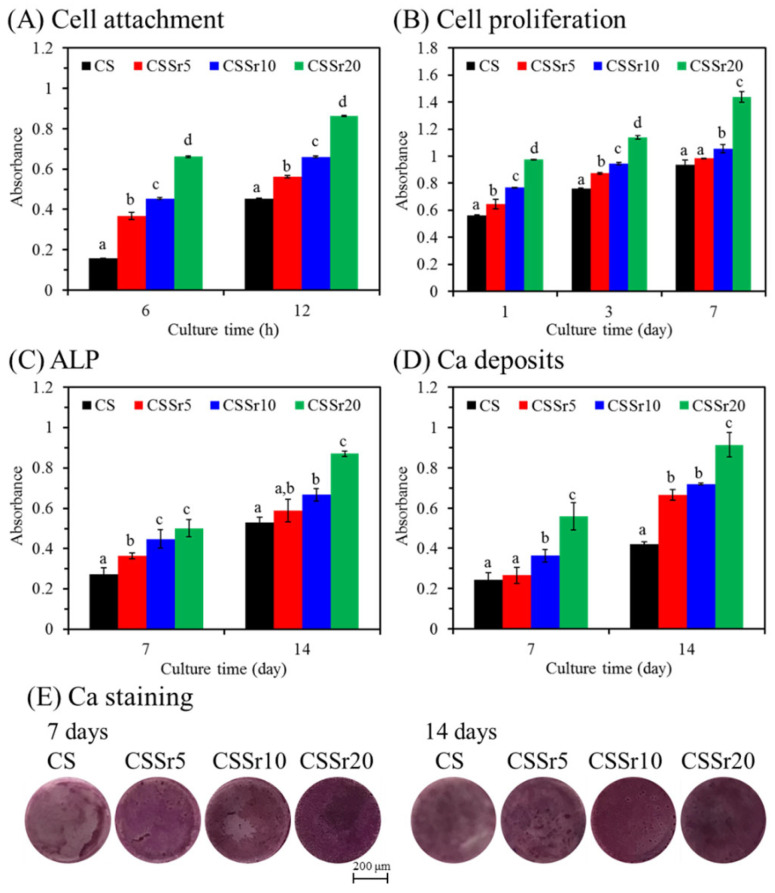
MG63 cells were cultured on the various sample surfaces for different time points. (**A**) cell attachment, (**B**) cell proliferation, (**C**) ALP, (**D**) quantification of calcium mineral deposits, and (**E**) Ca staining photograph. Different lowercase letters represent significant differences (*p* < 0.05) at the same culture time (n = 3).

The impact of Sr content in cement on the biological functions of MG63 cells was evaluated. Figure 5A showed that cell growth increased with higher Sr content in the cement during the culture periods of 6 and 12 h, with significant differences (*p* < 0.05). Similar results are observed with a longer culture duration, as illustrated in Figure 5B. For instance, on day one, the number of MG63 cells on the surfaces of CSSr5, CSSr10, and CSSr20 cements was approximately 15%, 36%, and 73% higher, respectively, compared to the CS control. After 7 days of culture, there was a significant (*p* < 0.05) enhancement of 54% in cell proliferation for CSSr20 compared to the CS control.

#### 3.4.2. Cell Differentiation and Mineralization

The intracellular ALP level was measured to assess the differentiation activity of MG63 cells, as depicted in Figure 5C. Remarkably, incorporating Sr into the cements consistently elevated ALP levels across all incubation periods. For example, on day 7, the ALP level for CSSr5, CSSr10, and CSSr20 samples was approximately 35%, 66%, and 85% higher than that of the CS control. The quantification and staining of calcium mineral deposits, evaluated through the Alizarin Red S assay, were illustrated in Figure 5D,E. After 7 days of culture, a significant increase (*p* < 0.05) in calcium content was observed in the CSSr20 cement compared to the CS control, showing a 1.6-fold increase (Figure 5D). By day 14, the calcium deposits in the cells continued to rise, with the calcium deposits on the CSSr20 cement surface being twice as much as those on the CS cement. Consistent with the quantitative findings, the calcium staining on cements with higher Sr content exhibited more intense staining than that on the control cement on both the 7th and 14th day, as illustrated in Figure 5E.

### 3.5. Antibacterial Ability

**Figure 6:**
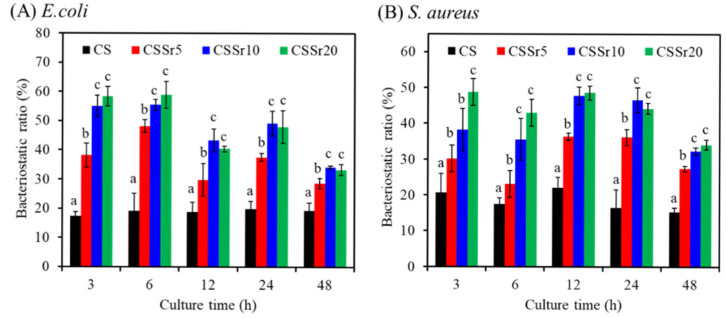
Bacteriostatic ratio (%) of various cements after (**A**) *E. coli* or (**B**) *S. aureus* culture in contact with the cements at short-time and long-time points. Different lowercase letters represent significant differences (*p* < 0.05) at the same culture time (n = 3).

We measured the bacteriostatic ratio (%) against *E. coli* and *S. aureus* to investigate the antimicrobial activity of Sr content in CS cement. As shown in Figure 6A, three hours after inoculation with *E. coli*, the cement surface displayed a bacteriostatic ratio that correlated with the Sr content. After six hours, the bacteriostatic ratios for CS, CSSr5, CSSr10, and CSSr20 increased to 19%, 48%, 55%, and 59%, respectively. At 12, 24, and 48 h, the bacteriostatic ratios of all Sr-containing cements were statistically significantly higher (*p* < 0.05) than those of the CS cement. Remarkably, after 48 h of *E. coli* exposure, the killing efficacy of CSSr10 and CSSr20 was 1.7 times greater than that of the control group. Furthermore, the study revealed that, when tested against *S. aureus*, the cements with higher Sr content consistently showcased significantly superior bacteriostatic ratios throughout the inoculation period (Figure 6B). At 48 h, both CSSr10 and CSSr20 exhibited bacteriostatic ratios more than twice that of the CS control. During this period, the antimicrobial activity of CSSr10 and CSSr20 was comparable against Gram-negative and Gram-positive microorganisms.

## 4. Discussion

To effectively address diverse clinical needs, it is essential to tailor the characteristics of bone cements by leveraging the material structure and chemistry of their components. This study explored the impact of varying Sr levels on calcium silicate bone cement, focusing on its physicochemical properties, mechanical strength, and biological functions. Regarding the powders, the results from XRD and FTIR analyses indicated that the incorporation of Sr had minimal impact on the phase composition of calcium silicate powders, as noted by Lin et al. [5]. However, at higher Sr concentrations, a minor presence of the CaSrSiO_4_ phase was detected, which was associated with a broad peak observed in the 31–33° range. It is well established that the radius of the Sr ion (1.13 Å) is slightly larger than that of the calcium (Ca) ion (1.00 Å) [31]. This slight size difference allowed Sr ions to be integrated into the crystalline lattice of calcium silicate cement, replacing Ca ions and resulting in a broadened diffraction peak. This phenomenon can be elucidated through the mechanisms of solid solution formation and the microstrain effect, corroborating findings from previous research [32,33]. Additionally, the morphologies of four calcium silicate-based powders revealed a similar heterogeneous structure composed of multiple phases. The ability of Sr to easily assimilate into the lattice structure underscores a significant opportunity for enhancing material properties through strategic ion substitutions, pointing towards a promising direction for advancing bone cement development.

The setting time of bone cement is a critical factor that can significantly impact its effectiveness in clinical applications. When the amount of Sr was increased in a calcium silicate cement, the setting time significantly extended from 17 min to 39 min, aligning with findings from previous studies [16,34]. This extended setting time is concerning, as the optimal range for clinical bone cement is between 10 and 25 min. Such delays can result in incompletely set bone cement paste, posing risks such as embolism in the surrounding bone tissue [4,35]. Although the addition of Sr slowed the setting reaction of calcium silicate cement, it can be incorporated within the C–S–H structure at different sites. Additionally, a small amount of Sr can replace calcium (Ca) in the CaO sheets through a Sr^2+^–Ca^2+^ ion exchange process [36,37]. Consequently, a CaSrSiO_4_ phase may form in the Sr-containing cement after hydration.

In addition to the prolonged setting time, low mechanical properties of calcium silicate cements may limit their clinical applications [38,39]. The initial diametral tensile strength of various cements ranged from 2.4 to 2.6 MPa, which was lower than that of commercial calcium silicate-based cements [8]. Although these bone cements are primarily used for low or non-load-bearing bone defects, such as those found in maxillofacial reconstructive surgeries and vertebroplasty [40,41], there is still room to improve the setting time and mechanical strength. Enhancements can be achieved by substituting trace elements, such as magnesium [38] or boron [39], or by adding chelating agents like phytic acid [42].

The smooth structure of the cement, made of fine particle agglomerates, can be primarily recognized as a hydration product. In addition, when the cement contained elevated levels of Sr, the gel structure revealed the presence of fibrils. Cement hydration plays a crucial role in connecting the originally hydrophilic particles, forming a colloidal gel with bonding properties [43].

The radiopacity of materials is a critical factor in ensuring effective post-observation. In this study, Sr was strategically used to significantly replace Ca, thereby enhancing radiopacity. Our results compellingly demonstrated that increasing the concentration of the Sr dopant directly correlated with improved radiopacity of the cement. This remarkable effect was due to Sr’s higher atomic number than Ca, allowing for superior absorption of X-ray energy. Notably, the cements containing 10% (4.6 mm Al) and 20% Sr (6.1 mm Al) exceeded the recommended radiopacity threshold of 3 mm Al (ISO 6876:2001 standards). Similarly, Romieu et al. developed a Sr-containing calcium phosphate bone cement that exhibited three times the radiopacity of cortical bone for vertebroplasty [17].

Evaluating the in vitro bioactivity of a material is essential because the formation of an apatite layer on its surface plays a pivotal role in establishing a direct bond with living bone. This interaction is a key factor in the success of biomedical implants and treatments, thereby underscoring the significance of in vitro bioactivity testing in material development [44]. Calcium silicate-based cement has been demonstrated to exhibit bioactivity in SBF [43,45]. Our developed bone cements effectively formed apatite spherulites on their surfaces within just one day of soaking in SBF, showcasing their high bioactivity. Furthermore, incorporating Sr into calcium silicate did not compromise its ability to form apatite in SBF, consistent with previous research [46].

Regarding changes in strength, the initial strength of the hardened cements increased after 7-day soaking in SBF. This increase was attributed to the continued hardening of the initial reactants [43]. During the powder/water mixing process, certain activated phases, such as β-Ca_2_SiO_4_, within the hardened cement remained partially unreacted, resulting in weaker interactions among cement particles. However, immersing the cement samples in SBF facilitated a more thorough hardening process, leading to a substantial increase in strength—an outcome supported by numerous studies [43,47]. Once the strength reached its maximum value after soaking for 14 days, a subsequent decline in strength was likely due to increased porosity.

Porosity and weight loss generally serve as degradation indicators and tend to increase with prolonged soaking times for degradable and porous materials. This rise in porosity is primarily due to water and ions from the solution penetrating the inner structure of the cement through existing defects and pores [43]. This increasing porosity can be harnessed by bone tissue cells to enhance the osteogenesis of the degradable biomaterial, facilitating its transformation into genuine bone tissue [48]. At earlier time points, such as after 7-day soaking, the four cement samples exhibited a relatively minor weight increase due to apatite formation. However, as time progressed, pronounced weight loss became increasingly evident. Notably, strength, porosity, and weight loss measurements revealed no substantial differences among the four types of calcium silicate-based cements. Furthermore, research by Zhang et al. concluded that Sr does not substantially affect the degradation rate of calcium silicate [49], further supporting the consistency of these findings.

Interactions between cells and cement materials are crucial for effective bone formation and significantly impact the success of these materials. Previous studies have demonstrated that Sr-containing materials can enhance osteoblastic cell viability and ALP activity, thereby stimulating bone formation [13,21,23,30,50,51]. ALP, a crucial membrane-bound exoenzyme, plays a vital role in the early stages of osteogenic differentiation during bone regeneration. Additionally, the ability of cells to generate mineralized matrices and nodules on material surfaces is critical for effective bone regeneration. In vitro analyses of this study consistently demonstrated that cells cultured on cement surfaces with increased strontium (Sr) content showed significantly higher viable cell counts, elevated alkaline phosphatase (ALP) activity, and greater calcium deposits. Zhang et al. highlighted that the Sr-incorporated mesoporous bioactive glass scaffolds promote osteogenic differentiation of osteoporotic bone marrow stromal cells in vitro and enhance bone regeneration in a rat model after ovariectomy [50]. Additionally, Lee et al. have identified Sr as a potent nanotherapeutic platform for implantable biomaterials, effectively addressing osteoporotic bone defects in an ovariectomized rat model [51]. These findings underscore the translational potential of Sr-containing materials in advancing bone regeneration therapies.

Developing bone cement with antibacterial properties is essential for preventing bacterial adhesion during the critical process of bone healing. Numerous studies have formulated various bone cements by incorporating antibiotics or antibacterial agents, significantly enhancing their effectiveness against bacteria [4,52]. Notably, the potential of Sr for strengthening the antibacterial activity of biomaterials has not been as thoroughly investigated as the well-established benefits for osteogenic properties. Our current research revealed that integrating Sr into calcium silicate cement amplifies its antibacterial efficacy. Alshammari et al. demonstrated that Sr(OH)_2_ exhibits potent, dose-dependent antibacterial properties against bacteria linked to peri-implantitis [28]. Furthermore, our recent discoveries indicated that Sr ions possess good antibacterial capabilities, primarily due to their ability to generate reactive oxygen species, which may play a crucial role in combating bacterial growth [27]. The compelling results from our study revealed the advantages of Sr-incorporated calcium silicate cement over conventional calcium silicate cement. This modification enhanced radiopacity, boosting antibacterial efficacy and osteogenic potential. Among the formulations tested, the 10% Sr-containing cement demonstrated excellent osteogenic performance, adequate radiopacity, and satisfactory setting time, positioning it as a potential candidate for clinical applications.

## 5. Conclusions

This study unveiled the significant effects of Sr on the radiopacity and antibacterial efficacy of calcium silicate cement. The findings indicated that the cement enriched with 10% Sr (CSSr10) exhibited a radiopacity of 4.6 mm Al, exceeding ISO standards. Higher amounts of Sr in the calcium silicate cement significantly promoted the proliferation and differentiation of MG63 cells. Importantly, the presence of Sr effectively inhibited the growth of two bacterial strains, *E coli* and *S. aureus*, in a concentration-dependent manner. With its suitable setting time, adequate radiopacity, excellent osteogenic activity, and potent antibacterial efficacy, the 10% Sr-enriched calcium silicate cement may show promise as a bone filler for applications in vertebroplasty, kyphoplasty, and endodontics.

## Figures and Tables

**Table 1:** Code, dopant content, setting time, diametral tensile strength, porosity, and radiopacity of various cements.

Code	Sr/(Sr + Ca) (mol%)	Setting Time (min)	Strength (MPa)	Porosity (%)	Radiopacity (mm Al)
CS	0	17 ± 2 ^a^	2.4 ± 0.4 ^a^	24 ± 3 ^a^	1.9 ± 0.2 ^a^
CSSr5	5	22 ± 2 ^b^	2.4 ± 0.5 ^a^	23 ± 2 ^a^	3.1 ± 0.3 ^b^
CSSr10	10	29 ± 2 ^c^	2.6 ± 0.4 ^a^	28 ± 5 ^a^	4.6 ± 0.3 ^c^
CSSr20	20	39 ± 2 ^d^	2.5 ± 0.4 ^a^	27 ± 3 ^a^	6.1 ± 0.3 ^d^

Mean values followed by the same superscript letters were not significantly different from CS.

## Data Availability

The original contributions presented in the study are included in the article, further inquiries can be directed to the corresponding author.
